# Anticancer activity of luteolin glycosides

**DOI:** 10.17179/excli2020-2747

**Published:** 2020-08-14

**Authors:** Ahmed Ghallab

**Affiliations:** 1 Forensic Medicine and Toxicology Department, Faculty of Veterinary Medicine, South Valley University, Qena, Egypt

## ⁯⁯⁯

***Dear Editor,***

Recently, Lee and colleagues published a study on cytostatic effects of luteolin glycosides in MDA-MB-231 breast cancer cells (Lee et al., 2019[10]). Luteolin and its derivate have been shown to inhibit migration of several cell lines (Kim et al., 2012[7]; 2018[8][9]; Park et al., 2013[14]). In the present study, the authors focused on MDA-MB-231 cells, a HER2-negative, as well as estrogen and progesterone receptor negative cell line, because triple-negative cancer cells represent a challenge in breast cancer therapy (Callmann et al., 2020[3]). The authors demonstrate that luteolin inhibited migration and invasion of MDA-MB-231 cells stimulated with the tumor promoter 12-O-tetradecanoylphorbol-3-acetate already at a non-cytotoxic concentration of 5 µM (Lee et al., 2019[10]). At cytotoxic concentrations luteoline caused Fas-mediated apoptosis (Lee et al., 2019[10]). 

Improved treatment options of triple negative breast cancer are urgently needed (Wang et al., 2020[20]; Moss et al., 2020[13]). Factors responsible for prognosis and metastasis of breast cancer include the cellular and humoral immune system (Schmidt et al., 2012[16], 2018[17]; Heimes et al., 2017[4][5]), cholin metabolism associated genes (Marchan et al., 2017[12]; Lesjak et al., 2014[11]; Stewart et al., 2012[18]), antioxidative factors (Cadenas et al., 2014[1], 2019[2]; Hellwig et al., 2016[6]), actin associated proteins (Stock et al., 2015[19]; Rommerswinkel et al., 2018[15]), and many more. It will be interesting to learn in future if luteolin glycosides, which show promising effects in breast cancer cell lines *in vitro* will also be efficient in mouse tumor models. 

## Conflict of interest

The authors declare no conflict of interest.
